# Supplementary material for: Body size prediction in scorpions: a phylogenetic comparative examination of linear measurements of individual body parts
Source: PeerJ. 2025 Jan 17;13:e18621. doi: 10.7717/peerj.18621 (PMC11745135; doi:10.7717/peerj.18621)
Supplement: Supplemental Information 1 [file peerj-13-18621-s001.pdf]

Supporting information 1

Body size prediction in scorpions: a phylogenetic comparative examination of linear measurements of individual body parts

Stênio Í. A. Foerster*

2024-11-06

Contents

Species List . . . 2
List of articles utilized for gathering morphological data . . . 7
ModelFinder results . . . 14
Calibration points . . . 15
R Function for defining lognormal priors . . . 18
Interaction models . . . 22
Phylogenetic multiple regressions . . . 25
References . . . 26

*Department of Zoology, University of Tartu, Estonia. Contact: stenio.foerster@ut.ee | stenioit@gmail.com

Species List

List of scorpion species included in the phylogenetic and statistical analyses. Accession codes for new sequences are in blue. For each species, the number of male and female individuals used to calculate species means is provided, with missing data ("-") for species included solely for reconstructing the phylogenetic tree.

Species	Females	Males	Total	COI	16S	18S	28S
Aegaeobuthus cyprius	1	1	2	DQ310849	DQ310812	-	-
Aegaeobuthus nigrocinctus	0	2	2	ON255557	ON262578	-	-
Alayotityus delacruzi	4	4	8	-	DQ990826	-	-
Alayotityus juraguaensis	1	0	1	-	DQ990827	-	-
Alayotityus nanus	2	0	2	-	DQ990828	-	-
Alayotityus sierramaestrae	1	0	1	-	KY674447	-	-
Ananteris ashaninka	1	1	2	ON255558	-	ON259096	ON259166
Ananteris balzanii	2	5	7	KY674491	KY674448	-	KY674470
Ananteris franckei	5	5	10	PQ341284	-	-	-
Ananteris mauryi	28	23	51	PQ341283	-	-	-
Ananteris platnicki	1	1	2	ON255559	ON262579	ON259097	ON259167
Androctonus amoreuxi	3	2	5	ON255560	ON262580	-	MW557083
Androctonus australis	3	2	5	ON255561	ON262581	ON259098	ON259168
Androctonus bicolor	2	3	5	KF548120	KJ538332	-	-
Androctonus bourdoni	0	1	1	ON255562	ON262582	-	-
Androctonus crassicauda	1	1	2	ON255563	ON262583	GQ335459	-
Androctonus gonneti	1	0	1	ON255564	ON262584	-	-
Androctonus hoggarensis	2	1	3	JN018150	-	JN018267	JN018364
Androctonus liouvillei	1	1	2	-	ON262585	-	ON259170
Androctonus mauritanicus	1	2	3	ON255565	ON262586	ON259099	ON259169
Androctonus sergenti	1	0	1	ON255566	ON262587	ON259100	ON259171
Babycurus gigas	1	2	3	ON255569	ON262590	ON259103	ON259174
Barbaracurus sofomarensis	1	2	3	ON255570	ON262591	ON259104	ON259175
Barbaracurus zambonellii	1	1	2	ON255571	ON262592	ON259105	ON259176
Buthacus occidentalis	0	1	1	JN018151	-	JN018268	JN018365
Buthacus stockmanni	1	1	2	ON255572	ON262593	ON259106	ON259177
Butheolus harrisoni	2	2	4	ON255573	ON262594	ON259107	ON259178
Buthoscorpio sarasinorum	1	0	1	ON255574	ON262595	ON259108	ON259179
Buthus albengai	1	0	1	JN885909	-	-	-
Buthus atlantis	1	1	2	ON255575	ON262596	ON259109	ON259180
Buthus awashensis	1	1	2	ON255576	ON262597	ON259110	ON259181
Buthus draa	1	1	2	ON255577	ON262598	-	-
Buthus elmoutaouakili	0	1	1	ON255578	ON262599	-	ON259182
Buthus lienhardi	1	1	2	ON255579	ON262600	-	-
Buthus malhommei	1	1	2	JN885935	-	-	-
Buthus mardochei	1	1	2	ON255580	ON262601	-	ON259183
Buthus maroccanus	2	0	2	ON255581	-	ON259111	ON259184
Buthus occitanus	1	2	3	ON255582	ON262602	-	-
Buthus paris	2	2	4	ON255583	ON262603	-	-
Centruroides arctimanus	1	1	2	MK479161	MK479008	MK479059	MK479110
Centruroides baergi	1	1	2	MK479162	MK479009	MK479060	ON259185
Centruroides balsasensis	1	1	2	MK479163	MK479010	MK479061	MK479112
Centruroides baracoae	6	6	12	ON255586	ON262606	ON259112	ON259187
Centruroides barbudensis	1	1	2	MK479165	MK479012	MK479063	MK479114

Continues on the next page

Species	Females	Males	Total	COI	16S	18S	28S
Centruroides borinquensis	1	1	2	MK479167	MK479014	MK479065	MK479116
Centruroides chamulaensis	2	2	4	MK479168	MK479015	MK479066	MK479117
Centruroides edwardsii	3	4	7	MK479169	MK479016	MK479067	MK479118
Centruroides elegans	1	1	2	MK479170	MK479017	MK479068	MK479119
Centruroides exilimanus	2	2	4	MK479171	MK479018	MK479069	MK479120
Centruroides farri	1	0	1	MK479172	MK479019	MK479070	MK479121
Centruroides flavopictus	0	3	3	MK479173	MK479020	MK479071	MK479122
Centruroides gracilis	5	4	9	KY982180	KY981894	KY981989	ON259189
Centruroides guanensis	3	3	6	MK479211	MK479058	MK479109	MK479160
Centruroides hoffmanni	2	1	3	MZ429061	MK479025	MK479076	ON259190
Centruroides infamatus	2	1	3	KY982181	KY981895	KY981990	MK479128
Centruroides limpidus	0	3	3	MK479184	MK479031	MK479082	MK479133
Centruroides luceorum	3	0	3	MK479185	MK479032	MK479083	MK479134
Centruroides lucidus	3	4	7	ON255590	ON262610	-	ON259191
Centruroides marcanoi	0	4	4	MK479186	MK479033	MK479084	ON259192
Centruroides meisei	6	6	12	MK479187	MK479034	MK479085	MK479136
Centruroides meridionalis	0	2	2	MK479189	MK479035	MK479086	MK479137
Centruroides nigrimanus	1	2	3	MK479191	MK479038	MK479089	MK479140
Centruroides nigrovariatus	1	2	3	MK479192	MK479039	MK479090	MK479141
Centruroides noxius	2	2	4	MK479193	MK479040	MK479091	MK479142
Centruroides ochraceus	2	2	4	MK479194	MK479041	MK479092	MK479143
Centruroides ornatus	3	2	5	MK479195	MK479042	MK479093	MK479144
Centruroides platnicki	3	2	5	MK479196	MK479043	MK479094	MK479145
Centruroides pococki	1	1	2	MK479197	MK479044	MK479095	MK479146
Centruroides rileyi	3	4	7	KY982183	KY981897	KY981992	KY982087
Centruroides robertoi	1	1	2	ON255592	ON262612	-	ON259193
Centruroides schmidti	7	8	15	KY982184	KY981898	KY981993	KY982089
Centruroides serrano	1	1	2	MK479200	MK479047	MK479098	MK479149
Centruroides simplex	1	1	2	MK479202	MK479048	MK479099	MK479154
Centruroides sissomi	1	0	1	MK479201	MK479049	MK479100	MK479150
Centruroides taino	4	4	8	MK479204	MK479051	MK479102	MK479152
Centruroides tapachulaensis	4	4	8	MK479205	MK479052	MK479103	MK479153
Centruroides testaceus	2	1	3	MK479207	MK479054	MK479105	MK479155
Centruroides thorellii	1	1	2	MK479208	MK479055	MK479106	MK479157
Centruroides tuxtla	1	1	2	MK479209	MK479056	MK479107	MK479158
Centruroides vittatus	0	1	1	KY982185	KY981899	KY981994	MK479159
Chaneke hofereki	1	2	3	ON255594	ON262613	ON259116	ON259195
Charmus laneus	2	0	2	ON255595	ON262614	ON259117	ON259196
Compsobuthus arabicus	1	1	2	-	AY226177	-	-
Compsobuthus eritreaensis	1	1	2	ON255597	ON262616	ON259118	ON259198
Compsobuthus somalilandus	1	1	2	ON255600	-	-	-
Femtobuthus shutuae	1	1	2	ON255601	ON262620	ON259119	ON259201
Gint amoudensis	2	2	4	OM722200	OM809158	-	-
Gint banfasae	1	2	3	OM722193	OM809151	-	-
Gint calviceps	0	1	1	-	OM809149	-	-
Gint dabakalo	1	1	2	OM722190	OM809147	-	-
Gint gaitako	1	1	2	OM722188	OM809145	ON259120	ON259202
Gint gubanensis	0	1	1	OM722182	OM809139	-	-
Gint maidensis	1	1	2	OM722180	OM809137	-	-
Grosphus madagascariensis	1	1	2	ON255602	ON262621	ON259121	ON259203
Heteroctenus abudi	4	3	7	KY982186	ON262622	KY981995	ON259204
Heteroctenus bonettii	4	3	7	KY982189	KY981903	KY981998	KY982094
Heteroctenus garridoi	1	1	2	KY982191	KY981905	KY982000	KY982096
Heteroctenus junceus	4	4	8	KY982198	KY981912	KY982007	KY982097
Heteroctenus princeps	3	4	7	KY982200	KY981914	KY982009	ON259205
Hottentotta flavidulus	2	0	2	ON255606	ON262625	-	ON259207

Continues on the next page

Species	Females	Males	Total	COI	16S	18S	28S
Hottentotta franzwerneri	0	1	1	JF820094	-	-	-
Hottentotta gentili	1	1	2	ON255607	ON262626	-	ON259208
Hottentotta hottentotta	1	0	1	ON255608	ON262627	ON259124	ON259209
Hottentotta tamulus	1	1	2	KT716032	-	-	-
Ischnotelson peruassu	1	1	2	KY982208	KY981922	KY982017	KY982112
Isometrus maculatus	11	5	16	KY982207	KY981921	KY982016	KY982111
Isometrus thwaitesi	1	0	1	ON255611	ON262630	ON259126	ON259212
Jaguajir agamemnon	1	0	1	KY982209	KY981923	KY982018	KY982113
Jaguajir pintoi	2	2	4	KY982220	KY981934	KY982029	KY982124
Jaguajir rochae	15	15	30	KY982224	KY981938	KY982033	KY982128
Karasbergia methueni	1	0	1	ON255613	ON262632	ON259128	ON259214
Leiurus abdullahbayrami	2	1	3	ON255614	ON262633	ON259129	ON259215
Leiurus quinquestriatus	3	2	5	JQ514258	JQ514241	-	-
Lissothus occidentalis	0	1	1	ON255615	ON262634	ON259130	ON259216
Lychas mucronatus	3	2	5	JN018153	AF370855	JN018270	JN018367
Lychas obsti	1	1	2	ON255616	ON262635	ON259131	ON259217
Lychas scutilus	1	1	2	ON255617	ON262636	-	ON259218
Mesobuthus afghanus	1	1	2	AJ550707	-	-	-
Mesobuthus eupeus	1	1	2	AJ550701	AY228141	-	-
Mesobuthus haarlovi	0	2	2	ON255618	ON262637	ON259132	ON259219
Mesobuthus philippovitschi	1	1	2	HM567348	-	-	-
Mesobuthus phillipsi	1	1	2	ON255619	ON262638	ON259133	ON259220
Mesobuthus thersites	1	1	2	AJ550700	-	-	-
Microbuthus gardneri	1	1	2	ON255620	ON262639	ON259134	ON259221
Microbuthus kristensenorum	2	0	2	ON255621	ON262640	ON259135	ON259222
Microbuthus satyrus	1	1	2	ON255622	ON262641	ON259136	ON259223
Microtityus consuelo	3	3	6	ON255623	ON262642	-	ON259224
Microtityus fundorai	1	1	2	-	DQ990830	-	-
Microtityus solegladi	2	2	4	ON255624	ON262643	ON259137	ON259225
Neobuthus awashensis	1	1	2	ON255625	ON262644	-	-
Neobuthus eritreaensis	1	1	2	ON255626	ON262645	ON259138	ON259226
Neobuthus kutcheri	2	2	4	OM722176	OM809133	-	-
Odontobuthus brevidigitus	1	1	2	ON255627	ON262646	ON259139	ON259227
Odontobuthus tavighiae	1	1	2	KF701324	KJ659599	-	-
Odontobuthus tirgari	1	1	2	KF701322	KJ659602	-	-
Olivierus caucasicus	0	2	2	MG586931	MG586916	-	-
Olivierus elenae	1	0	1	AJ783604	MG586917	-	-
Olivierus gorelovi	1	1	2	AJ783515	-	-	-
Olivierus intermedius	1	1	2	MG586940	MG586926	-	-
Olivierus kreuzbergi	1	1	2	AJ550697	MG586929	-	-
Olivierus martensii	1	1	2	DQ340065	-	FJ948787.1	FJ948787
Olivierus nenilini	1	1	2	AJ783522	-	-	-
Orthochirus afganus	1	0	1	ON255630	ON262649	-	-
Orthochirus innesi	3	1	4	ON255632	ON262651	ON259142	ON259230
Parabuthus kajibu	1	1	2	ON255634	ON262653	ON259144	ON259232
Physoctonus debilis	23	15	38	KY982238	KY981952	KY982048	KY982143
Physoctonus striatus	0	2	2	KY982241	KY981955	KY982051	KY982146
Picobuthus wahibaensis	1	1	2	ON255636	ON262655	ON259145	ON259234
Reddyanus basilicus	0	1	1	ON255638	ON262658	ON259148	ON259237
Reddyanus ceylonensis	1	1	2	ON255639	ON262659	-	ON259238
Reddyanus petrzelkai	1	1	2	ON255641	ON262661	ON259150	ON259240
Rhopalurus caribensis	1	1	2	KY982243	KY981957	KY982053	KY982147
Rhopalurus laticauda	4	7	11	KY982247	KY981961	KY982057	KY982149
Rhopalurus ochoai	3	3	6	MF508635	MF402014	MF508621	MF508628
Somalicharmus whitmanae	0	1	1	ON255642	ON262662	ON259151	ON259241
Thaicharmus mahunkai	2	0	2	ON255644	ON262664	ON259153	ON259243

Continues on the next page

Species	Females	Males	Total	COI	16S	18S	28S
Tityus argentinus	1	1	2	KY674493	KY674452	-	KY674474
Tityus atriventer	0	1	1	KY982264	KY981978	KY982074	-
Tityus bahiensis	1	1	2	KY982265	KY981979	KY982075	OK493233
Tityus blaseri	1	1	2	OK561901	OK493248	-	OK493221
Tityus brazilae	7	1	8	PQ341280	OK493239	-	OK493225
Tityus cf. stigmurus SF247	4	6	10	PQ341281	-	-	-
Tityus cf stigmurus SF464	0	3	3	PQ341282	-	-	-
Tityus clathratus	1	5	6	KY982266	KY981980	KY982076	KY982171
Tityus confluens	2	2	4	KY674496	KY674456	-	KY674478
Tityus crassimanus	0	2	2	ON255647	ON262667	ON259156	ON259246
Tityus curupi	1	1	2	KY674440	KY674424	-	KY674429
Tityus discrepans	1	1	2	KY982267	KY981981	KY982077	KY982172
Tityus elii	1	2	3	-	ON262669	-	ON259248
Tityus forcipula	2	2	4	OK561898	OK493251	-	OK493230
Tityus gaffini	1	1	2	ON255649	ON262670	-	ON259249
Tityus kuryi	11	2	13	KY982268	KY981982	KY982078	KY982173
Tityus mattogrossensis	2	2	4	KY674498	KY674458	-	KY674480
Tityus neglectus	1	4	5	PQ341279	-	-	-
Tityus neibae	1	1	2	ON255650	ON262671	ON259158	ON259250
Tityus nematochirus	1	1	2	OL416036	-	-	OL416386
Tityus obscurus	0	1	1	OK561905	OK493243	-	OK493229
Tityus panguana	1	1	2	OK561908	OK493244	-	OK493231
Tityus paraguayensis	1	1	2	KY674499	-	-	-
Tityus pictus microdon	1	2	3	KY982271	KY981985	KY982081	KY982176
Tityus potameis	1	1	2	OK561899	OK493245	-	OK493232
Tityus pusillus	30	30	60	PQ341278	-	KP276456	KP276376
Tityus serrulatus	3	2	5	OK561900	OK493247	-	OK493220
Tityus smithii	2	2	4	ON255651	ON262672	ON259159	ON259251
Tityus spelaeus	17	0	17	-	OK493249	-	OK493224
Tityus stigmurus	10	4	14	OK561896	OK493240	-	OK493227
Tityus uruguayensis	1	1	2	KY674445	KY674428	-	KY674484
Troglorhopalurus lacrau	1	0	1	MF508640	MF508619	MF508626	MF508632
Troglorhopalurus translucidus	4	2	6	MF508641	MF508620	MF508627	MF508634
Trypanothacus barnesi	1	1	2	ON255652	ON262673	ON259160	ON259252
Vachoniolus globimanus	1	1	2	-	AY226179	-	-
Xenobuthus xanthus	2	1	3	ON255657	ON262679	ON259164	ON259258
Zabius birabeni	1	1	2	KY982272	KY981986	KY982082	KY674485
Zabius fuscus	1	1	2	KY982273	KY981987	KY982083	KY674486
Aegaeobuthus gibbosus	-	-	-	ON255556	ON262577	ON259095	ON259165
Ananteris sp. R033MACr35702	-	-	-	KY674492	KY674449	-	KY674471
Androctonus sp.	-	-	-	ON255567	ON262588	ON259101	ON259172
Apistobuthus pterygocercus	-	-	-	ON255568	ON262589	ON259102	ON259173
Centruroides bani	-	-	-	MK479164	ON262605	MK479062	ON259186
Centruroides bicolor	-	-	-	MK479166	MK479013	MK479064	MK479115
Centruroides exilicauda	-	-	-	KY982179	KY981893	KY981988	KY982084
Centruroides fulvipes	-	-	-	MK479174	MK479021	MK479072	ON259188
Centruroides griseus	-	-	-	MK479176	MK479023	MK479074	MK479125
Centruroides hentzi	-	-	-	MK479177	MK479024	MK479075	MK479126
Centruroides insulanus	-	-	-	MK479180	MK479027	MK479079	MK479129
Centruroides insularis	-	-	-	MK479181	MK479028	MK479078	MK479130
Centruroides koesteri	-	-	-	MK479182	MK479029	MK479080	MK479131
Centruroides limbatus	-	-	-	MK479183	MK479030	MK479081	MK479132
Centruroides morenoi	-	-	-	MK479188	MK479036	MK479087	MK479138
Centruroides nigrescens	-	-	-	MK479190	MK479037	MK479088	MK479139
Centruroides pallidiceps	-	-	-	AY995836	-	-	-
Centruroides sasae	-	-	-	MK479198	MK479045	MK479096	MK479147

Continues on the next page

Species	Females	Males	Total	COI	16S	18S	28S
Centruroides sculpturatus	-	-	-	MK479199	MK479046	MK479097	MK479148
Centruroides suffusus	-	-	-	MK479203	MK479050	MK479101	MK479151
Centruroides tecomanus	-	-	-	MK479206	MK479053	MK479104	MK479156
Compsobuthus acutecaritus	-	-	-	ON255596	ON262615	-	ON259197
Compsobuthus maindroni	-	-	-	ON255598	ON262617	-	ON259199
Compsobuthus matthiesseni	-	-	-	ON255599	ON262618	-	ON259200
Compsobuthus sp.	-	-	-	-	ON262619	-	-
Hottentotta conspersus	-	-	-	ON255605	ON262624	ON259123	ON259206
Hottentotta jabalpurensis	-	-	-	MK696578	-	-	-
Hottentotta jayakari	-	-	-	-	AY226176	-	-
Hottentotta minusalta	-	-	-	ON255609	ON262628	-	ON259210
Hottentotta pachyurus	-	-	-	KT716023	-	-	-
Hottentotta rugiscutis	-	-	-	KT716025	-	-	-
Hottentotta trilineatus	-	-	-	ON255610	ON262629	ON259125	ON259211
Hottentotta vinchu	-	-	-	MH820411	-	-	-
Ischnotelson guambiensis	-	-	-	KY982205	KY981919	KY982014	KY982109
Janalychas srilankensis	-	-	-	ON255612	ON262631	ON259127	ON259213
Janalychas tricaritus	-	-	-	KT716037	-	-	-
Odontobuthus bidentatus	-	-	-	KF701316	KJ659596	-	-
Odontobuthus doriae	-	-	-	KF701313	KJ659608	-	-
Olivierus fuscus	-	-	-	MG586937	MG586921	-	-
Olivierus kaznakovi	-	-	-	ON255628	ON262647	ON259140	ON259228
Olivierus longichelus	-	-	-	PP229938	-	-	-
Olivierus parthorum	-	-	-	AJ783516	-	-	-
Orthochiroides vachoni	-	-	-	ON255629	ON262648	ON259141	ON259229
Orthochirus bicolor	-	-	-	KT716038	-	-	-
Orthochirus fomichevi	-	-	-	MT232527	-	-	-
Orthochirus glabrifrons	-	-	-	ON255631	ON262650	-	-
Orthochirus iranus	-	-	-	-	MK170441	-	-
Parabuthus brevimanus	-	-	-	ON255633	ON262652	ON259143	ON259231
Parabuthus laevifrons	-	-	-	JN018154	-	JN018271	JN018368
Parabuthus pallidus	-	-	-	ON255635	ON262654	-	ON259233
Parabuthus transvaalicus	-	-	-	JQ514247	JQ514229	-	-
Reddyanus loebli	-	-	-	ON255640	ON262660	ON259149	ON259239
Teruelius flavopiceus	-	-	-	ON255643	ON262663	ON259152	ON259242
Thaicharmus sp.	-	-	-	ON255645	ON262665	ON259154	ON259244
Tityopsis inexpectata	-	-	-	ON255646	ON262666	ON259155	ON259245
Tityus carvalhoi	-	-	-	KY674495	KY674455	-	-
Tityus cf. bastosi	-	-	-	KY674494	KY674454	-	KY674476
Tityus charreyroni	-	-	-	OK561903	OK493237	-	OK493222
Tityus ecuadoriensis	-	-	-	ON255648	ON262668	ON259157	ON259247
Tityus metuendus	-	-	-	KY982269	KY981983	KY982079	KY982174
Tityus riverai	-	-	-	KY982270	KY981984	KY982080	-
Tityus sastrei	-	-	-	OK561897	OK493252	-	OK493234
Tityus soratensis	-	-	-	KY674500	KY674460	-	KY674482
Tityus trivittatus	-	-	-	KY674501	KY674461	-	-
Uroplectes emiliae	-	-	-	ON255653	ON262674	-	ON259253
Uroplectes fischeri	-	-	-	ON255654	ON262675	ON259161	ON259254
Uroplectes flavoviridis	-	-	-	-	ON262676	ON259162	ON259255
Uroplectes gracilior	-	-	-	ON255655	ON262677	ON259163	ON259256
Uroplectes otjimbinguensis	-	-	-	ON255656	ON262678	-	ON259257
Zabius sp. 1 R007MACr36495	-	-	-	KY674505	KY674465	-	KY674487
Zabius sp. 2 R010MACr36496	-	-	-	KY674506	KY674466	-	KY674488
Zabius sp. 3 R034MACr36497	-	-	-	KY674507	KY674467	-	KY674489
Zabius sp. 4 R035MACr36498	-	-	-	KY674508	KY674468	-	KY674490
Brachistosternus paposo	-	-	-	KT446990	KT446655	KT446767	KT446879

List of articles utilized for gathering morphological data

Amr, Z. S., & El-Oran, R. (1994). Systematics and distribution of scorpions (Arachnida, Scorpionida) in Jordan. Italian Journal of Zoology, 61(2), 185–190.

Armas, L. de. (1996). Presencia de Centruroides schmidti Sissom en el sureste de México y descripción de dos especies nuevas (Scorpiones: Buthidae). Revista Nicaraguense de Entomología, 36, 21–33.

Armas, L. de. (2001). Centruroides platnicki Armas, 1981 (Scorpiones: Buthidae): First record from Turks Islands, Bahamas. Revista Ibérica de Aracnología, 4, 27–28.

Armas, L. de, Luna-Sarmiento, D., & Flórez, E. (2012). Composición del género Centruroides Marx, 1890 (Scorpiones: Buthidae) en Colombia, con la descripción de una nueva especie. Boletín de La Sociedad Entomológica Aragonesa (S.E.A.), 50, 105–114.

Armas, L. de, & Martín-Frías, E. (1999). Complementos a la taxonomía de Centruroides nigrovariatus y C. elegans (Scorpiones: Buthidae) de México. Anales de La Escuela Nacional de Ciencias Biológicas, 44, 27–35.

Armas, L. F. (1973). Escorpiones del Archipielago Cubano: Nuevo Género y Nuevas Especies de Buthidae (Archinida: Scorpionida). Poeyana, 114, 1–28.

Armas, L. F. (1974a). Escorpiones del archipiélago cubano. II. Hallazgo del género Microtityus (Scorpionida: Buthidae), con las descripciones de un nuevo subgénero y tres nuevas especies. Poeyana, 132, 1–26.

Armas, L. F. (1974b). Escorpiones del Archipielago Cubano IV. Nuevas especies de Rhopalurus (Scorpionida: Buthidae). Poeyana, 136, 1–12.

Armas, L. F. (1976). Escorpiones del Archipielago Cubano, V: Nuevas especies de Centruroides (Scorpionida; Buthidae). Poeyana, 146, 1–55.

Armas, L. F. (1981). El genero Centruroides Marx, 1889 (Scorpiones: Buthidae), en Bahamas y Republica Dominicana. Poeyana, 223, 1–21.

Armas, L. F. (1999a). Nueva especie de Centruroides (Scorpiones: Buthidae) de Chiapas, México. Novitates Caribaea, 1, 47–52.

Armas, L. F. (1999b). Quince nuevos alacranes de La Española y Navassa, Antillas Mayores (Arachnida: Scorpiones). Avicennia, 10(11), 101–136.

Armas, L. F. (2002). Alacranes de República Dominicana. Centruroides nitidus (Thorell, 1876) y Microtityus lantiguai Armas & Marcano Fondeur, 1992 (Scorpiones: Buthidae). Revista Ibérica de Aracnología, 5, 61–66.

Armas, L. F., & Antun, A. J. A. (2004). Adiciones al género" Tityus" CL Koch, 1836 en República Dominicana, con la descripción de dos especies nuevas (Scorpiones: Buthidae). Revista Ibérica de Aracnología, 10, 53–64.

Armas, L. F., Beutelspacher, C. R., & Martin, E. F. (1995). Notas sobre la taxonomia y distribuicion de algunos Centruroides (Scorpiones: Buthidae) de Mexico. Revista Nicaraguense de Entomología, 32, 29–43.

Armas, L. F., & Frías, E. M. (2008). El género Centruroides Marx, 1890 (Scorpiones: Buthidae) en el estado de Veracruz, México. Boletín de La Sociedad Entomológica Aragonesa (S.E.A.), 43, 7–22.

Armas, L. F., & Marcano-Fondeur, E. J. (1987). Nuevos escorpiones (Arachnida: Scorpiones) de República Dominicana. Poeyana, 356, 1–28.

Armas, L. F., & Marcano-Fondeur, E. J. (1992). Nuevos alacranes de República Dominicana (Arachnida: Scorpiones). Poeyana, 420, 1–36.

Armas, L. F., & Teruel, R. (2012). Revisión del género Microtityus Kjellesvig-Waering, 1966 (Scorpiones: Buthidae) en República Dominicana. Revista Ibérica de Aracnología, 21, 69–88.

Armas, L. F., Teruel, R., & Kovařík, F. (2011a). On Centruroides margaritatus (Gervais, 1841) and closely related species (Scorpiones: Buthidae). Euscorpius, 2011(132), 1–16.

Armas, L. F., Teruel, R., & Kovařík, F. (2011b). Redescription of Centruroides granosus (Thorell, 1876) and identity of Centrurus granosus simplex Thorell, 1876 (Scorpiones: Buthidae). Euscorpius, 2011(127), 1–11.

Armas, L. F., Trujillo, R. E., Viquez, C., & Agreda, E. O. (2010). Primer registro de Centruroides tapachulaensis Hoffmann, 1932 (Scorpiones: Buthidae) para Guatemala. Boletín de La Sociedad Entomológica Aragonesa (S.E.A.), 46, 261–266.

Baldazo-Monsivaiz, J. G., Ponce-Saavedra, J., & Flores-Moreno, M. (2013). Una especie nueva de alacrán del género Centruroides de importancia médica (Scorpiones: Buthidae) del estado de Guerrero, México. Revista Mexicana de Biodiversidad, 84(1), 100–116.

Bohorquez Gomez, R. M. (2016). Revisión taxonómica y distribución geográfica de las especies de Tityus, Subgénero Atreus,(Scorpiones, Buthidae) presentes en Colombia [Master Thesis]. Universidad Nacional de Colombia.

Botero-Trujillo, R. (2008). Redescription of Tityus (Tityus) gaffini Lourenço, 2000 (Scorpiones: Buthidae) and new locality records. Revista Ibérica de Aracnología, 16, 29–34.

Coelho, P., Kaliontzopoulou, A., Rasko, M., & van der Meijden, A. (2017). A 'striking'relationship: Scorpion defensive behaviour and its relation to morphology and performance. Functional Ecology, 31(7), 1390–1404.

Deshpande, S., Joshi, M., Kawai, K., Deb, A., Lee, J.-D., Bastawade, D., Gowande, G., & Sulakhe, S. (2023). Molecular and morphological confirmation of Isometrus maculatus (DeGeer, 1778)(Scorpiones: Buthidae) from Northeast India and East Asia. Euscorpius, 2023(374), 1–19.

Di, Z.-Y., Cao, Z.-J., Wu, Y.-L., Zhu, L., Liu, H., & Li, W.-X. (2013). The scorpions of Hainan Island, China (Arachnida: Scorpiones). Euscorpius, 2013(153), 1–22.

Esposito, L. A., Yamaguti, H. Y., Souza, C. A., Pinto-Da-Rocha, R., & Prendini, L. (2017). Systematic revision of the neotropical club-tailed scorpions, Physoctonus, Rhopalurus, and Troglorhopalurus, revalidation of Heteroctenus, and descriptions of two new genera and three new species (Buthidae: Rhopalurusinae). Bulletin of the American Museum of Natural History, 2017(415), 1–136.

Fet, V., Hendrixson, B. E., Sissom, W. D., & Levy, G. (2000). First record for the genus Mesobuthus Vachon, 1950 in Israel: Mesobuthus nigrocinctus (Ehrenberg, 1828), n. Comb. (Scorpiones: Buthidae) from Mt. Hermon. Israel Journal of Ecology and Evolution, 46(4), 287–295.

Fet, V., Kovařík, F., Gantenbein, B., Kaiser, R. C., Graham, M. R., & Stewart, A. K. (2018). Revision of the Mesobuthus caucasicus complex from Central Asia, with descriptions of six new species (Scorpiones: Buthidae). Euscorpius, 255, 1–77.

Gallão, J. E., & Bichuette, M. E. (2016). On the enigmatic troglobitic scorpion Troglorhopalurus translucidus: Distribution, description of adult females, life history and comments on Rhopalurus lacrau (Scorpiones: Buthidae). Zoologia (Curitiba), 33, e20150193.

Gantenbein, B., Kropf, C., Largiader, C. R., Scholl, A., & others. (2000). Molecular and morphological evidence for the presence of a new buthid taxon (Scorpiones: Buthidae) on the island of Cyprus. Revue Suisse de Zoologie, 107(1), 213.

Goodman, A. M., Prendini, L., Francke, O. F., & Esposito, L. A. (2021). Systematic revision of the arboreal Neotropical "thorellii" clade of Centruroides Marx, 1890, bark scorpions (Buthidae CL Koch, 1837) with descriptions of six new species. Bulletin of the American Museum of Natural History, 452(1), 1–92.

Heddergott, M., Stubbe, M., Stubbe, W., Steinbach, P., & Stubbe, A. (2016). Geographical distribution of the genus Mesobuthus (Scorpiones: Buthidae) in Mongolia. Erforschung Biologischer Ressourcen Der Mongolei, 13, 147–164.

Khalil, N., & Yağmur, E. A. (2010). Leiurus abdullahbayrami (Scorpiones: Buthidae), a new species for the scorpion fauna of Syria. Serket, 12(1), 1–6.

Kovařík, F. (1997). Revision of the genera Lychas and Hemilychas, with descriptions of six new species (Scorpiones: Buthidae). Acta Societatis Zoologicae Bohemicae, 61, 311–371.

Kovařík, F. (2006). Review of Tunisian species of the genus Buthus with descriptions of two new species and a discussion of Ehrenberg's types (Scorpiones: Buthidae). Euscorpius, 2006(34), 1–16.

Kovařík, F. (2011). Buthus awashensis sp. N. From Ethiopia (Scorpiones: Buthidae). Euscorpius, 2011(128), 1–6.

Kovařík, F., Fet, V., Gantenbein, B., Graham, M. R., Yağmur, E. A., Šťáhlavský, F., Poverennyi, N. M., & Novruzov, N. E. (2022). A revision of the genus Mesobuthus Vachon, 1950, with a description of 14 new species (Scorpiones: Buthidae). Euscorpius, 348.

Kovařík, F., & Lowe, G. (2012). Review of the genus Neobuthus Hirst, 1911 with description of a new species from Ethiopia (Scorpiones: Buthidae). Euscorpius, 2012(138), 1–25.

Kovařík, F., & Lowe, G. (2019). Scorpions of the Horn of Africa (Arachnida, Scorpiones). Part XVIII. Gint banfasae sp. N. From Somaliland (Buthidae). Euscorpius, 2019(272), 1–14. https://doi.org/10.18590/euscorpius.2019.vol2019.iss272.1

Kovařík, F., Lowe, G., Just, P., Ibrahim Awale, A., Sh Abdirahman Elmi, H., & Šťáhlavský, F. (2018). Scorpions of the Horn of Africa (Arachnida: Scorpiones). Part XV. Review of the genus Gint Kovařík et al., 2013, with description of three new species from Somaliland (Scorpiones, Buthidae). Euscorpius, 2018(259), 1–41.

Kovařík, F., Lowe, G., Plíšková, J., & Šťáhlavský, F. (2013). A new scorpion genus, Gint gen. N., from the Horn of Africa (Scorpiones: Buthidae). Euscorpius, 173, 1–19.

Kovařík, F., Lowe, G., Plíšková, J., & Šťáhlavský, F. (2016a). Scorpions of the Horn of Africa (Arachnida: Scorpiones). Part VI. Compsobuthus Vachon, 1949 (Buthidae), with a description of C. eritreaensis sp. N. Euscorpius, 2016(226), 1–21.

Kovařík, F., Lowe, G., Plíšková, J., & Šťáhlavský, F. (2016b). Scorpions of the Horn of Africa (Arachnida: Scorpiones). Part VII. Parabuthus Pocock, 1890 (Buthidae), with description of P. hamar sp. N. And P. kajibu sp. N. From Ethiopia. Euscorpius, 228, 1–58.

Kovařík, F., Lowe, G., Ranawana, K. B., Hoferek, D., Jayarathne, V. S., Plíšková, J., & Šťáhlavský, F. (2016). Scorpions of Sri Lanka (Arachnida, Scorpiones: Buthidae, Chaerilidae, Scorpionidae) with description of four new species of the genera Charmus Karsch, 1879 and Reddyanus Vachon, 1972 stat. N. Euscorpius, 220, 1–133.

Kovařík, F., Lowe, G., Seiter, M., Plíšková, J., & Šťáhlavský, F. (2015). Scorpions of Ethiopia (Arachnida: Scorpiones). Part II. Genus Babycurus Karsch, 1886 (Buthidae), with description of two new species. Euscorpius, 196, 1–31.

Kovařík, F., Lowe, G., & Šťáhlavský, F. (2016). Review of northwestern African Buthacus, with description of Buthacus stockmanni sp. N. From Morocco and Western Sahara (Scorpiones, Buthidae). Euscorpius, 236, 1–18.

Kovařík, F., Lowe, G., & Šťáhlavský, F. (2018). Review of the genus Babycurus Karsch, 1886 (Arachnida, Scorpiones, Buthidae), with descriptions of Barbaracurus gen. N. And two new species from Oman and Yemen. Euscorpius, 2018(267), 1–41.

Kovařík, F., Lowe, G., Stockmann, M., & Šťáhlavský, F. (2020). Notes on Compsobuthus Vachon, 1949: Redescription of C. arabicus Levy et al., 1973 from Arabia, and description of two new species from North Africa (Scorpiones: Buthidae). Euscorpius, 2020(298), 1–40.

Kovařík, F., & Mazuch, T. (2015). Review of the genus Gint Kovařík et al., 2013, with description of two new species from Somaliland and Somalia (Puntland) (Scorpiones: Buthidae). Euscorpius, 209, 1–23.

Kovařík, F., Teruel, R., & Lowe, G. (2016). Two new scorpions of the genus Chaneke Francke, Teruel et Santibáñez-López, 2014 (Scorpiones: Buthidae) from southern Mexico. Euscorpius, 2016(218), 1–20.

Kovařík, F., Teruel, R., Lowe, G., & Friedrich, S. (2015). Four new scorpion species (Scorpiones: Buthidae) from Amazonian Peru. Euscorpius, 210, 1–40.

Lenarducci, Â. R. I., Pinto-da-Rocha, R., & Lucas, S. M. (2005). Descrição de uma nova espécie de Rhopalurus thorell, 1876 (scorpiones: Buthidae) do Nordeste Brasileiro. Biota Neotropica, 5, 173–180.

Lira, A. F., Foerster, S. I. A., Albuquerque, C. M., & Moura, G. J. (2021). Contrasting patterns at interspecific and intraspecific levels in scorpion body size across a climatic gradient from rainforest to dryland vegetation. Zoology, 146, 125908.

Lourenço, W. R. (1979). Le scorpion Buthidae: Tityus mattogrossensis Borelli, 1901.(Morphologie, écologie, biologie et développement postembryonnaire). Bulletin Du Museum National d'Histoire Naturelle, 4(1), 95–117.

Lourenço, W. R. (1982). Revision du genre Ananteris Thorell, 1891 (Scorpiones, Buthidae), et description de six especes nouvelles. Bulletin Du Museum National d'Histoire Naturelle. Section A. Zoologie, Biologie et Ecologie Animales, 4(1).

Lourenço, W. R. (1983). Contribution a la connaissance de Centruroides barbudensis (Pocock 1898) (Scorpiones, Buthidae). Journal of Arachnology, 327–335.

Lourenço, W. R. (2003). Compléments à la faune de scorpions (Arachnida) de l'Afrique du Nord, avec des considérations sur le genre Buthus Leach, 1815. Revue Suisse de Zoologie, 110(4), 875–912.

Lourenço, W. R. (2008). The geographic pattern of distribution of the genus Rhopalurus Thorell, 1876 in the Guayana-Amazon region (Scorpiones: Buthidae). Euscorpius, 2008(73), 1–14.

Lourenço, W. R. (2012a). Further considerations on Tityus (Archaeotityus) clathratus CL Koch, 1844 and description of two associated new species (Scorpiones: Buthidae). Boletín de La Sociedad Entomológica Aragonesa (S.E.A.), 50, 277–283.

Lourenço, W. R. (2012b). Further taxonomic considerations on the genus buthoscorpio Werner, 1936 (Scorpiones, Buthidae), with description of a new species from India. Boletín de La Sociedad Entomológica Aragonesa (S.E.A.), 50, 187–192.

Lourenço, W. R. (2012c). The genus Ananteris thorell, 1891 (scorpiones, buthidae) in the Northeast region of Brazil and description of a new species. Boletín de La Sociedad Entomológica Aragonesa (S.E.A.), 50, 73–76.

Lourenço, W. R. (2014). The genus Rhopalurus Thorell, 1876 (Scorpiones: Buthidae) in northeast Brazil; a possible case of a vicariant species. Acta Biológica Paranaense (Curitiba), 43(1–2), 69–76.

Lourenço, W. R. (2020). A possible relict population of Mesobuthus (Aegaeobuthus?) nigrocinctus (Ehrenberg, 1828) in the Bishri Mountains of Syria (Scorpiones: Buthidae). Serket, 17(2).

Lourenço, W. R. (2021). Some additional comments on the Ananterinae or Ananteris group and description of a new species of Ananteris Thorell, 1891 from Central Brazil (Scorpiones: Buthidae). Zootaxa, 4984(1), 347–356.

Lourenço, W. R., Baptista, R. L. C., & Giupponi, A. P. L. (2004). Troglobitic scorpions: A new genus and species from Brazil. Comptes Rendus Biologies, 327(12), 1151–1156.

Lourenço, W. R., Giupponi, A. P., & Leguin, E.-A. (2013). Description of three more new species of the genus Ananteris. Anais Da Academia Brasileira de Ciências, 85, 709–725.

Lourenço, W. R., Jesus-Junior, M., & Limeira-de-Oliveira, F. (2006). A new species of Tityus CL Koch, 1836 (Scorpiones, Buthidae) from the state of Maranhão in Brazil. Boletín de La Sociedad Entomológica Aragonesa (S.E.A.), 38(1), 117–120.

Lourenço, W. R., & Leguin, E.-A. (2008). The true identity of Scorpio (Atreus) obscurus Gervais, 1843 (Scorpiones, Buthidae). Euscorpius, 2008(75), 1–9.

Lourenço, W. R., Modry, D., & Amr, Z. (2002). Description of a new species of Leiurus Ehrenberg, 1828 (Scorpiones, Buthidae) from the south of Jordan. Revue Suisse de Zoologie, 109(3), 635–642.

Lourenço, W. R., & Pinto-Da-Rocha, R. (1997). A reappraisal of the geographic distribution of the genus Rhopalurus Thorell (Scorpiones, Buthidae) and description of two new species. Biogeographica, 73(4), 181–191.

Lourenço, W. R., & Sadine, S. E. (2021). The genus Orthochirus Karsch, 1891, in Algeria with description of a new species (Scorpiones, Buthidae). Bulletin de La Société Entomologique de France, 126(2), 175–181.

Lourenço, W. R., & Vachon, M. (2004). Considérations sur le genre Buthus Leach, 1815 en Espagne, et description de deux nouvelles espèces (Scorpiones, Buthidae). Revista Ibérica de Aracnología, 9, 81–94.

Lourenço, W. R., & von Eickstedt, V. R. D. (1984). Descrição de uma espécie nova de Tityus coletada no Estado da Bahia, Brasil (Scorpiones, Buthidae). Journal of Arachnology, 55–60.

Lourenço, W. R., & von Eickstedt, V. R. D. (1988). Sinopse das espécies de Tityus do Nordeste do Brasil, com a redescrição da T. neglectus Mello-Leitão (Scorpiones, Burthidae). Revista Brasileira de Zoologia, 5, 399–408.

Lourenço, W. R., & Ythier, E. (2013). The remarkable scorpion diversity in the Ecuadorian Andes and description of a new species of Tityus CL Koch, 1836 (Scorpiones, Buthidae). ZooKeys, 307, 1.

Lourenço, W. R., & Ythier, E. (2017). Description of Tityus (Atreus) cisandinus sp. N. From Ecuadorian Amazonia, with comments on some related species (Scorpiones: Buthidae). Arachnida-Rivista Aracnologica Italiana, 15, 18–34.

Lourenço, W. R., Ythier, E., & Leguin, E.-A. (2009). A new species of Androctonus Ehrenberg, 1828 from Morocco (Scorpiones: Buthidae). Euscorpius, 2009(89), 1–8.

Lourenço, W., & Silva, E. (2007). New evidence for a disrupted distribution pattern of the Tityus confluens complex, with the description of a new species from the State of Para, Brazil (Scorpiones, Buthidae). Amazoniana, 19(3/4), 77–86.

Lowe, G. (2024). Pers. Comm.

Lowe, G. (2010a). A new species of Odontobuthus (Scorpiones: Buthidae) from northern Oman. Euscorpius, 2010(96), 1–22.

Lowe, G. (2010b). New picobuthoid scorpions (Scorpiones: Buthidae) from Oman. Euscorpius, 2010(93), 1–53.

Lowe, G. (2010c). The genus Vachoniolus (Scorpiones: Buthidae) in Oman. Euscorpius, 2010(100), 1–37.

Lowe, G. (2018a). The genera Butheolus Simon, 1882 and Xenobuthus gen. Nov. (Scorpiones: Buthidae) in Oman. Euscorpius, 261, 1–73.

Lowe, G. (2018b). The genera Butheolus Simon, 1882 and Xenobuthus gen. Nov.(Scorpiones: Buthidae) in Oman. Euscorpius, 261, 1–73.

Lowe, G., & Kovařík, F. (2016). Scorpions of the Horn of Africa (Arachnida, Scorpiones). Part V. Two new species of Neobuthus Hirst, 1911 (Buthidae), from Ethiopia and Eritrea. Euscorpius, 224, 1–46.

Lowe, G., Kovařík, F., Stockmann, M., & Št'áhlavskỳ, F. (2018). Review of Microbuthus with Description of M. satyrus sp. N.(Scorpiones, Buthidae) from Oman and Yemen. Euscorpius, 2018(263), 1–22.

Lowe, G., Kovařík, F., Stockmann, M., & Št'áhlavskỳ, F. (2019). Trypanothacus gen. N., a new genus of burrowing scorpion from the Arabian Peninsula (Scorpiones: Buthidae). Euscorpius, 2019(277), 1–30.

Martín-Frías, E., de Armas, L. F., & Paniagua-Solís, J. F. (2005). Redescription of the Mexican scorpion Centruroides hoffmanni Armas, 1996 (Scorpiones: Buthidae). Euscorpius, 2005(22), 1–7.

Miranda, R. J., Bermúdez, S., Flórez, E., & Armas, L. F. (2020). A new species of tityus from panama and costa rica previously identified as tityus pachyurus pocock, 1897 (scorpiones: Buthidae). Revista Ibérica de Aracnología, 37, 197–204.

Montoya, M., & Armas, L. F. (2002). Escorpiones (Arachnida) del Archipiélago de Bocas del Toro, Panamá. Revista de Biología Tropical, 50(1), 155–160.

Moreno-González, J. A., Pinto-da-Rocha, R., & Gallão, J. E. (2021). Bringing order to a complex system: Phenotypic and genotypic evidence contribute to the taxonomy of Tityus (Scorpiones, Buthidae) and support the description of a new species. ZooKeys, 1075, 33.

Obuid-Allah, A. H., El-Shimy, N. A., Mahbob, M. A., Ali, R. S., & others. (2020). Survey and morphological studies on scorpions inhabiting New Valley Governorate, Egypt. Egyptian Academic Journal of Biological Sciences, B. Zoology, 12(2), 227–238.

Ojanguren Affilastro, A., Kochalka, J., Guerrero-Orellana, D., Garcete-Barrett, B., De Roodt, A., Borges, A., & Ceccarelli, F. S. (2021). Redefinition of the identity and phylogenetic position of Tityus trivittatus Kraepelin 1898, and description of Tityus carrilloi n. Sp. (Scorpiones; Buthidae), the most medically important scorpion of southern South America. Revista Del Museo Argentino de Ciencias Naturales, 23, 27–55. https://doi.org/10.22179/REVMACN.23.714

Ojanguren-Affilastro, A. A. (2005). Estudio monográfico de los escorpiones de la República Argentina. Revista Ibérica de Aracnología, 11, 75–241.

Ojanguren-Affilastro, A. A., Adilardi, R. S., Cajade, R., Ramirez, M. J., Ceccarelli, F. S., & Mola, L. M. (2017). Multiple approaches to understanding the taxonomic status of an enigmatic new scorpion species of the genus Tityus (Buthidae) from the biogeographic island of Paraje Tres Cerros (Argentina). PloS One, 12(7), e0181337.

Ozkan, O., Adiguzel, S., & Kar, S. (2006). Parametric values of Androctonus crassicauda (Oliver, 1807) (Scorpiones: Buthidae) from Turkey. Journal of Venomous Animals and Toxins Including Tropical Diseases, 12, 549–559.

Ponce Saavedra, J., & Francke, O. F. (2004). Una nueva especie de alacrán del género Centruroides Marx (1890) (Scorpionbes, Buthidae) de la depresión del Balsas, México. Acta Zoológica Mexicana, 20(2), 221–232.

Ponce-Saavedra, J., Quijano-Ravell, A. F., Teruel, R., & Francke, O. F. (2015). Redescription of Centruroides ornatus Pocock, 1902 (Scorpiones Buthidae), a montane scorpion from central Mexico. Revista Ibérica de Aracnología, 15, 81–89.

Prendini, L. (2001). Further additions to the scorpion fauna of Trinidad and Tobago. The Journal of Arachnology, 29(2), 173–188.

Prendini, L., Esposito, L. A., Huff, J. C., & Volschenk, E. S. (2009). Redescription of Rhopalurus abudi (Scorpiones, Buthidae), with first description of the male and first record from mainland Hispaniola. The Journal of Arachnology, 37(2), 206–224.

Qi, J.-X., Zhu, M.-S., & Lourenço, W. R. (2004). Redescription of mesobuthus martensii martensii (karsch, 1879)(scorpiones: Buthidae) from China. Revista Ibérica de Aracnología, 10, 137–144.

Rojas-Runjaic, F. J. M., & Armas, L. F. (2007). Dos nuevas especies venezolanas del grupo Tityus clathratus y notas sobre Tityus ramirezi Esquivel de Verde, 1968 (Scorpiones: Buthidae). Boletín de La Sociedad Entomológica Aragonesa (S.E.A.), 41, 53–66.

Seiter, M., & Turiel, C. (2013). First record of Androctonus australis (Linnaeus, 1758) from Jordan (Scorpiones: Buthidae). Revista Ibérica de Aracnología, 23, 95–98.

Sissom, W. D. (1995). Redescription of the scorpion Centruroides thorelli Kraepelin (Buthidae) and description of two new species. Journal of Arachnology, 91–99.

Sissom, W. D., & Francke, O. F. (1983). Redescription of Centruroides testaceus (DeGeer) and description of a new species from the Lesser Antilles (Scorpiones: Buthidae). Occasional Papers Museum Texas Tech University, 88, 1–14.

Sissom, W. D., & Lourenço, W. R. (1987). The genus Centruroides in South America (Scorpiones, Buthidae). Journal of Arachnology, 11–28.

Stockmann, M., Turiel, C., Althoff, F., Lowe, G., & Kovařík, F. (2016). First report of Lissothus occidentalis Vachon, 1950 (Scorpiones: Buthidae) from Morocco and Western Sahara, with notes on ecology and captive breeding. Euscorpius, 2016(235), 1–12.

Sun, D., & Sun, Z.-N. (2011). Notes on the genus Mesobuthus (Scorpiones: Buthidae) in China, with description of a new species. The Journal of Arachnology, 39(1), 59–75.

Teruel, R. (2000). Taxonomía del complejo Centruroides anchorellus Armas, 1976 (Scorpiones: Buthidae). Revista Ibérica de Aracnología, 1, 3–12.

Teruel, R. (2001). Redescripción de Alayotityus delacruzi Armas, 1973 (Scorpiones: Buthidae). Revista Ibérica de Aracnología, 3, 17–24.

Teruel, R. (2011). Redescription of Tityus pictus Pocock, 1893 and Tityus smithii Pocock, 1893, with notes on the Tityus species from the Lesser Antilles (Scorpiones: Buthidae). Euscorpius, 2011(125), 1–26.

Teruel, R., & Armas, L. F. (2006). Revisión del grupo "Tityus crassimanus" (Scorpiones: Buthidae). Boletín Sociedad Entomológica Aragonesa, 1(39), 139–149.

Teruel, R., & Armas, L. F. (2012). Redescripción de Rhopalurus junceus (Herbst 1800)(Scorpiones: Buthidae). Boletín de La Sociedad Entomológica Aragonesa (S.E.A.), 50, 153–174.

Teruel, R., & Cozijn, M. A. (2013). On the distribution of the genus Rhopalurus Thorell, 1876 (Scorpiones: Buthidae) in the southern Caribbean islands. Euscorpius, 2013(179), 1–7.

Teruel, R., de Armas, L. F., & Kovařík, F. (2015). A new species of Centruroides Marx, 1890 (Scorpiones: Buthidae) from southern Hispaniola, Greater Antilles. Euscorpius, 198, 1–18.

Teruel, R., & Kovařík, F. (2011). Redescription and taxonomic position of Tityus atriventer Pocock, 1897 (Scorpiones: Buthidae). Euscorpius, 2011(115), 1–9.

Teruel, R., & Kovařík, F. (2014). Redescription of Androctonus bicolor Ehrenberg, 1828, and Description of Androctonus turieli sp. N. From Tunisia (Scorpiones: Buthidae). Euscorpius, 186, 1–15.

Teruel, R., Kovařík, F., Lowe, G., & Friedrich, S. (2017). Complements to the taxonomy of some Amazonian scorpions (Scorpiones: Buthidae). Euscorpius, 245, 1–7.

Teruel, R., Ponce-Saavedra, J., & Quijano-Ravell, A. F. (2015). Redescription of Centruroides noxius and description of a closely related new species from western Mexico (Scorpiones: Buthidae). Revista Mexicana de Biodiversidad, 86(4), 896–911.

Teruel, R., & Rein, J. O. (2010). A new Hottentotta Birula, 1908 from Afghanistan, with a note on the generic position of Mesobuthus songi Lourenço, Qi et Zhu, 2005 (Scorpiones: Buthidae). Euscorpius, 2010(94), 1–8.

Teruel, R., & Roncallo, C. A. (2008). Rare or poorly known scorpions from Colombia. III. On the taxonomy and distribution of Rhopalurus laticauda Thorell, 1876 (Scorpiones: Buthidae), with description of a new species of the genus. Euscorpius, 2008(68), 1–12.

Teruel, R., & Stockwell, S. A. (2002). A revision of the scorpion fauna of Honduras, with the description of a new species (Scorpiones: Buthidae, Diplocentridae). Revista Ibérica de Aracnología, 6, 111–127.

Teruel, R., & Tietz, A. K. (2008). The true identity of Rhopalurus pintoi Mello-Leitão, 1932, with notes on the status and distribution of Rhopalurus crassicauda Caporiacco, 1947 (Scorpiones: Buthidae). Euscorpius, 2008(70), 1–14.

Turiel, C. (2014). A new species of Hottentotta Birula, 1908 (Scorpiones: Buthidae) from southern Morocco. Euscorpius, 2014(181), 1–9.

Vachon, M. (1952). Etudes sur les Scorpions. Institut Pasteur d'Algérie.

Viquez, C., & Armas, L. F. (2005). Primeros registros de Centruroides exilimanus Teruel & Stockwell, 2001 (Scorpiones: Buthidae) para Guatemala y El Salvador, con la descripción de la hembra adulta. Boletín de La Sociedad Entomológica Aragonesa (S.E.A.), 1(37), 169–170.

Ythier, E. (2023). A new species of Buthus Leach, 1815 from the Atlantic coast of Morocco (Scorpiones: Buthidae). Faunitaxys, 11(69), 1–7.

Ythier, E., & Lourenço, W. R. (2022). A new species of Androctonus Ehrenberg, 1828 from Western Sahara (Scorpiones: Buthidae). Serket, 18(3).

ModelFinder results

Best-fit DNA partition scheme obtained with ModelFinder and employed in IQ-TREE for estimating tree topology and branch lengths. PI = number of parsimony-informative sites, IS = number of invariant sites.

Partition	Number of taxa	Length (bp)	PI	IS	Model
18S rRNA	154	1752	115	1554	TNe+G4
28S rRNA	196	235	50	171	TNe+G4
16S rRNA (mtDNA)	234	265	169	79	GTR+F+G4
COI 1st position	253	195	75	110	TN+F+G4
COI 2nd position	253	195	24	146	GTR+F+G4
COI 3rd position	253	194	191	1	GTR+F+G4

Calibration points

Calibration points used for node age estimation in buthid scorpions are detailed below. Each label corresponds to a calibration point, as shown on the phylogeny illustrated on the following page. Minimum and maximum node ages were applied to convert the maximum likelihood tree, generated using IQ-TREE v.2.3.2, into an ultrametric tree through penalized maximum likelihood. Node ages in the resulting ultrametric tree were then estimated using Bayesian inference in BEAST v1.10.4, employing lognormal priors. The 2.5% and 97.5% quantiles (Qs) of these priors aligned with the minimum and maximum ages for each calibration point.

		Node age (My)			Prior specification		
Label	Split	Min.	Max.	Source	meanlog	sdlog	2.5%-97.5% Qs
a	Buthus group	44.00	53.29	Ojanguren-Affilastro et al. 2017	3.88	0.049	44.07-53.46
b	Ananteris sp./A. balzanii	2.12	7.20	Ojanguren-Affilastro et al. 2017	1.36	0.310	2.14-7.4
c	Greater Antilles clade of Centrutoides (fossil record)	17.00	20.00	Esposito and Prendini 2019	2.91	0.040	17.02-20.06
d	Greater Antilles/Chortis Maya clade of Centruroides	23.00	26.00	Esposito and Prendini 2019	3.20	0.030	23.02-26.06
e	Chortis Maya clade of Centruroides (fossil record)	15.00	23.00	Esposito and Prendini 2019	2.92	0.110	15.06-23.17
f	Centruroides/Heteroctenus	26.00	30.00	Esposito and Prendini 2019	3.33	0.040	26.03-30.07
g	Tityus group	26.04	42.17	Ojanguren-Affilastro et al. 2017	3.50	0.120	26.15-42.52
h	Tityus (Archeotityus) group	7.16	15.89	Ojanguren-Affilastro et al. 2017	2.37	0.200	7.21-16.11
i	Zabius spp.	3.29	7.38	Ojanguren-Affilastro et al. 2017	1.60	0.210	3.31-7.48

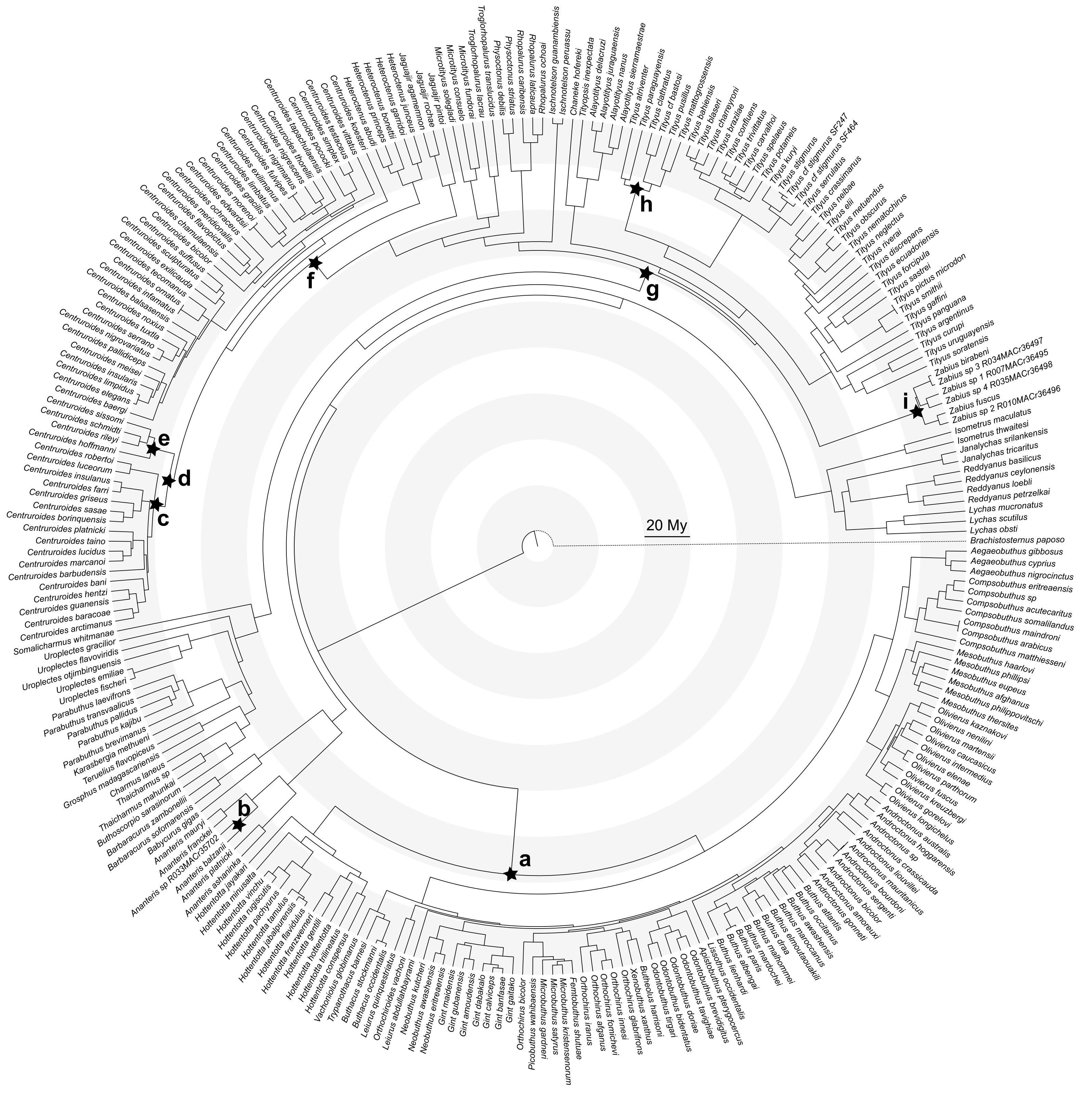
20 My
a
b
c
d
e
f
g
h
i
Brachistosternus paposo
Aegaeobuthus gibbosus
Aegaeobuthus cyprius
Aegaeobuthus nigrocinctus
Compsobuthus eritreaensis
Compsobuthus sp
Compsobuthus acutecaritus
Compsobuthus somalilandus
Compsobuthus maindroni
Compsobuthus arabicus
Compsobuthus matthiesseni
Mesobuthus haarlovi
Mesobuthus phillipsi
Mesobuthus eupeus
Mesobuthus afghanus
Mesobuthus philippovitschi
Mesobuthus thersites
Olivierus kaznakovi
Olivierus nenilini
Olivierus martensii
Olivierus caucasicus
Olivierus intermedius
Olivierus elenae
Olivierus parthorum
Olivierus fuscus
Olivierus kreuzbergi
Olivierus gorelovi
Olivierus longichelus
Androctonus australis
Androctonus hoggarensis
Androctonus sp
Androctonus crassicauda
Androctonus sp
Androctonus liouvillei
Androctonus mauritanicus
Androctonus bourdoni
Androctonus sergenti
Androctonus bicolor
Androctonus amoreuxi
Androctonus gonneti
Buthus atlantis
Buthus occitanus
Buthus awashensis
Buthus maroccanus
Buthus elmoutaouakili
Buthus draa
Buthus malhommei
Buthus mardochei
Buthus paris
Buthus albengai
Buthus lienhardi
Buthus occidentalis
Lissothus pterygocercus
Apistobuthus brevidigitus
Odontobuthus tavighiae
Odontobuthus doriae
Odontobuthus bidentatus
Odontobuthus tirgari
Butheolus harrisoni
Xenobuthus xanthus
Orthochirus glabrifrons
Orthochirus innesi
Orthochirus fomichevi
Orthochirus afganus
Orthochirus iranus
Femtobuthus shutuae
Microbuthus kristensenorum
Microbuthus satyrus
Microbuthus gardneri
Picobuthus wahibaensis
Orthochirus bicolor
Gint gaitako
Gint banfasae
Gint calviceps
Gint dabakalo
Gint amoudensis
Gint gubanensis
Gint maidensis
Neobuthus eritreaensis
Neobuthus awashensis
Neobuthus kutcheri
Leiurus abdullahbayrami
Orthochiroides vachoni
Leiurus quinquestriatus
Buthacus occidentalis
Buthacus stockmanni
Trypanothacus barnesi
Vachoniolus globimanus
Hottentotta conspersus
Hottentotta trilineatus
Hottentotta hottentotta
Hottentotta gentili
Hottentotta franzwerneri
Hottentotta flavidulus
Hottentotta jabalpurensis
Hottentotta tamulus
Hottentotta pachyurus
Hottentotta rugiscutis
Hottentotta vinchu
Hottentotta minusalta
Hottentotta jayakari
Ananteris ashaninka
Ananteris platnicki
Ananteris balzanii
Ananteris sp R033MACr35702
Ananteris franckei
Ananteris mauryi
Babycurus gigas
Barbaracurus sofomarensis
Barbaracurus zambonellii
Buthoscorpio sarasinorum
Thaicharmus mahunkai
Thaicharmus sp
Charmus laneus
Grosphus madagascariensis
Teruelius flavopiceus
Karasbergia methueni
Parabuthus brevimanus
Parabuthus kajibu
Parabuthus pallidus
Parabuthus transvaalicus
Parabuthus laevifrons
Uroplectes fischeri
Uroplectes emiliae
Uroplectes otjimbinguensis
Uroplectes flavoviridis
Uroplectes gracilior
Somalicharmus whitmanae
Centruroides arctimanus
Centruroides baracoae
Centruroides guanensis
Centruroides hentzi
Centruroides bani
Centruroides barbudensis
Centruroides marcanoi
Centruroides lucidus
Centruroides taino
Centruroides platnicki
Centruroides borinquensis
Centruroides sasae
Centruroides griseus
Centruroides farri
Centruroides insulanus
Centruroides luceorum
Centruroides robertoi
Centruroides hoffmanni
Centruroides rileyi
Centruroides schmidti
Centruroides sissomi
Centruroides baergi
Centruroides elegans
Centruroides limpidus
Centruroides insularis
Centruroides meisei
Centruroides pallidiceps
Centruroides nigrovariatus
Centruroides serrano
Centruroides tuxtla
Centruroides noxius
Centruroides balsasensis
Centruroides infamatus
Centruroides tecomanus
Centruroides ornatus
Centruroides exilicauda
Centruroides sculpturatus
Centruroides suffusus
Centruroides bicolor
Centruroides chamulaensis
Centruroides flavopictus
Centruroides meridionalis
Centruroides ochraceus
Centruroides edwardsii
Centruroides morenoi
Centruroides gracilis
Centruroides limbatus
Centruroides exilimanus
Centruroides fulvipes
Centruroides nigrimanus
Centruroides nigrescens
Centruroides thorellii
Centruroides tapachulaensis
Centruroides pococki
Centruroides simplex
Centruroides testaceus
Centruroides vittatus
Heteroctenus koesteri
Heteroctenus abudi
Heteroctenus princeps
Heteroctenus bonettii
Heteroctenus garridoi
Heteroctenus junceus
Jaguajir agamemnon
Jaguajir rochae
Jaguajir pintoi
Microtityus soleglad
Microtityus consuelo
Microtityus fundorai
Troglorhopalurus lacrau
Troglorhopalurus translucidus
Physoctonus debilis
Physoctonus striatus
Rhopalurus caribensis
Rhopalurus laticauda
Rhopalurus ochoai
Ischnotelson guanambiensis
Ischnotelson peruassu
Chaneke hofereki
Tityopsis inexpectata
Alayotityus delacruzi
Alayotityus juraguaensis
Alayotityus nanus
Alayotityus sierramaestrae
Tityus atriventer
Tityus paraguayensis
Tityus clathratus
Tityus cf bastosi
Tityus pusillus
Tityus mattogrossensis
Tityus bahiensis
Tityus blaseri
Tityus charreyroni
Tityus brazilae
Tityus confluens
Tityus trivittatus
Tityus carvalhoi
Tityus spelaeus
Tityus potameis
Tityus kuryi
Tityus stigmurus
Tityus cf stigmurus SF247
Tityus cf stigmurus SF464
Tityus serrulatus
Tityus crassimanus
Tityus neibae
Tityus elii
Tityus metuendus
Tityus obscurus
Tityus nematochirus
Tityus neglectus
Tityus riverai
Tityus discrepans
Tityus ecuadoriensis
Tityus forcipula
Tityus sastrei
Tityus pictus microdon
Tityus smithii
Tityus gaffini
Tityus panguana
Tityus argentinus
Tityus curupi
Tityus uruguayensis
Tityus soratensis
Zabius birabeni
Zabius sp 3 R034MACr36497
Zabius sp 1 R007MACr36495
Zabius sp 4 R035MACr36498
Zabius fuscus
Zabius sp 2 R010MACr36496
Isometrus maculatus
Isometrus thwaitesi
Janalychas srilankensis
Janalychas tricaritus
Reddyanus basilicus
Reddyanus ceylonensis
Reddyanus loebli
Reddyanus petrzelkai
Lychas mucronatus
Lychas scutilus
Lychas obsti

Maximum likelihood tree

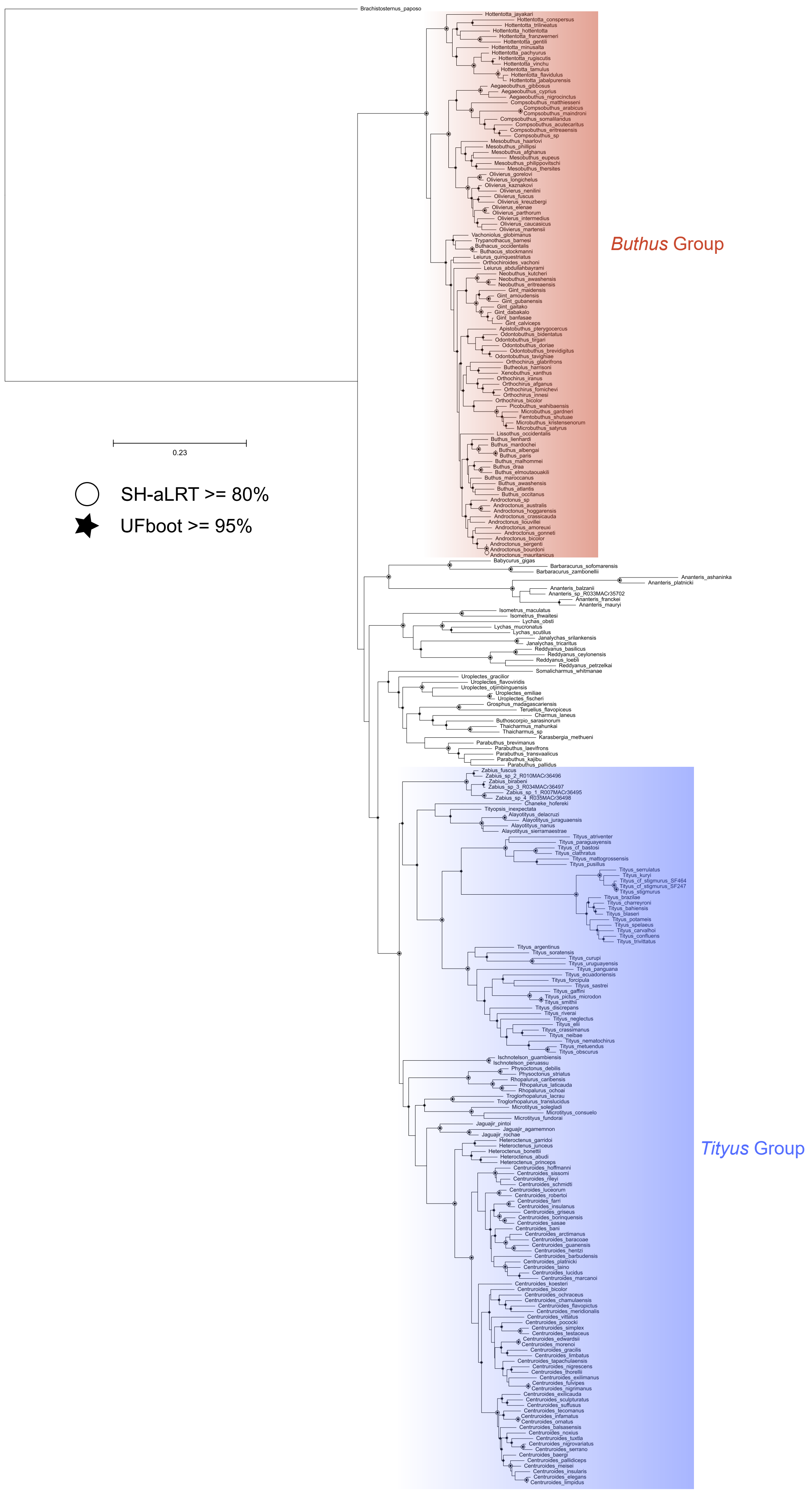

R Function for defining lognormal priors

The `get_lognormal` R function is designed to define lognormal priors for node age estimation in BEAST. It performs parameter optimization using the L-BFGS-B protocol to find the `meanlog` (μ) and `sdlog` (σ) parameters of a lognormal distribution, ensuring that the 2.5% and 97.5% quantiles align with the target quantiles – i.e., the prior belief about the respective node age. The objective function within `get_lognormal` aims to minimize the squared difference between the target quantiles and the actual quantiles for the given `meanlog` and `sdlog` values. It is defined as:

$$f(\mu, \sigma) = \sum_{i=1}^{n} (Q(p_i; \mu, \sigma) - T_i)^2$$

where:

- $f(\mu, \sigma)$ is the objective function,
- $Q(p_i; \mu, \sigma) = \exp(\mu + \sigma \cdot \Phi^{-1}(p_i))$ is the lognormal quantile function,
- μ is the `meanlog` (logarithmic mean of the distribution),
- σ is the `sdlog` (logarithmic standard deviation),
- p_i are the quantile probabilities (e.g., 0.025 and 0.975),
- T_i are the target quantiles (see the example below),
- $\Phi^{-1}(p)$ is the inverse of the CDF of the standard normal distribution.

Instructions

Simply copy the code provided on the next page and paste it into your R console. Press Enter, and you will see the `get_lognormal` function appear in your Environment panel (assuming you are using RStudio).

```r
# Function to find optimal values for meanlog and sdlog

get_lognormal <- function(target_quantiles, quantile_probs, seed_number) {

  # Define the objective function
  objective_function <- function(params) {
    meanlog <- params[1]
    sdlog <- params[2]

    # Calculate the actual quantiles for the given meanlog and sdlog
    actual_quantiles <- qlnorm(quantile_probs, meanlog, sdlog)

    # Calculate the sum of squared differences between target and actual quantiles
    sum((actual_quantiles - target_quantiles)^2)
  }

  # Set initial guesses for meanlog and sdlog
  initial_guess <- c(log((target_quantiles[1] + target_quantiles[2]) / 2), 0.5)

  # Use optim to find the best meanlog and sdlog
  result <- optim(initial_guess,
    objective_function,
    method = "L-BFGS-B",
    lower = c(-Inf, 0.01),
    upper = c(Inf, Inf)
  )

  # Extract the optimal meanlog and sdlog
  optimal_meanlog <- result$par[1]
  optimal_sdlog <- result$par[2]

  # Generate samples using the optimal parameters
  set.seed(seed_number)
  n_samples <- 1000
  samples <- rlnorm(n_samples, optimal_meanlog, optimal_sdlog)

  # Return the results

  return(
    cat(
      "Optimal meanlog:", round(optimal_meanlog, 3), "\n",
      "Optimal sdlog:", round(optimal_sdlog, 3), "\n",
      "Quantiles of 1000 simulated values:",
      round(quantile(samples, probs = quantile_probs), 3)
    )
  )
}
```

Usage

Let's say we have a fossil record for a specific node in our phylogeny, with an estimated age between 12 and 16 million years. We want to set a lognormal prior for this node so that the 2.5% and 97.5% quantiles of the lognormal distribution approximate this age range. In other words, we aim for the referred quantiles to be as close as possible to 12 and 16 million years. To achieve this, we will use the `get_lognormal` function to determine the `meanlog` and `sdlog` parameters of the lognormal distribution. The main arguments in the `get_lognormal` function are `target_quantiles`, which should include the minimum and maximum ages from our fossil record (12 and 16 million years in this case), and `quantile_probs`, which represent the quantiles of the lognormal distribution that should correspond to the values provided in `target_quantiles`. We also need to provide a seed number to ensure reproducibility (it can be any number). Thus, all we need to do is the following:

```
get_lognormal(
  target_quantiles = c(12, 16),
  quantile_probs = c(0.025, 0.975),
  seed_number = 10
)
```

```
## Optimal meanlog: 2.629
##  Optimal sdlog: 0.073
##  Quantiles of 1000 simulated values: 12.03 16.079
```

The function returns the optimal values for `meanlog` (2.629) and `sdlog` (0.073) for a lognormal distribution, ensuring that the 2.5% and 97.5% quantiles closely match the values provided via `target_quantiles`. We can generate and plot a lognormal distribution using the optimal `meanlog` and `sdlog`:

```
a <- rlnorm(n = 1000, meanlog = 2.629, sdlog = 0.073)
plot(density(a), main = NA, las = 1)
abline(v = 12, col = "red")
abline(v = 16, col = "red")
abline(v = 12.03, col = "blue", lty = "dashed")
abline(v = 16.079, col = "blue", lty = "dashed")
legend("topright",
  legend = c("Actual", "Estimated\nquantiles"),
  col = c("red", "blue"),
  lty = c("solid", "dashed"),
  bty = "n", cex = 0.8,
  y.intersp = 2.5
)
```

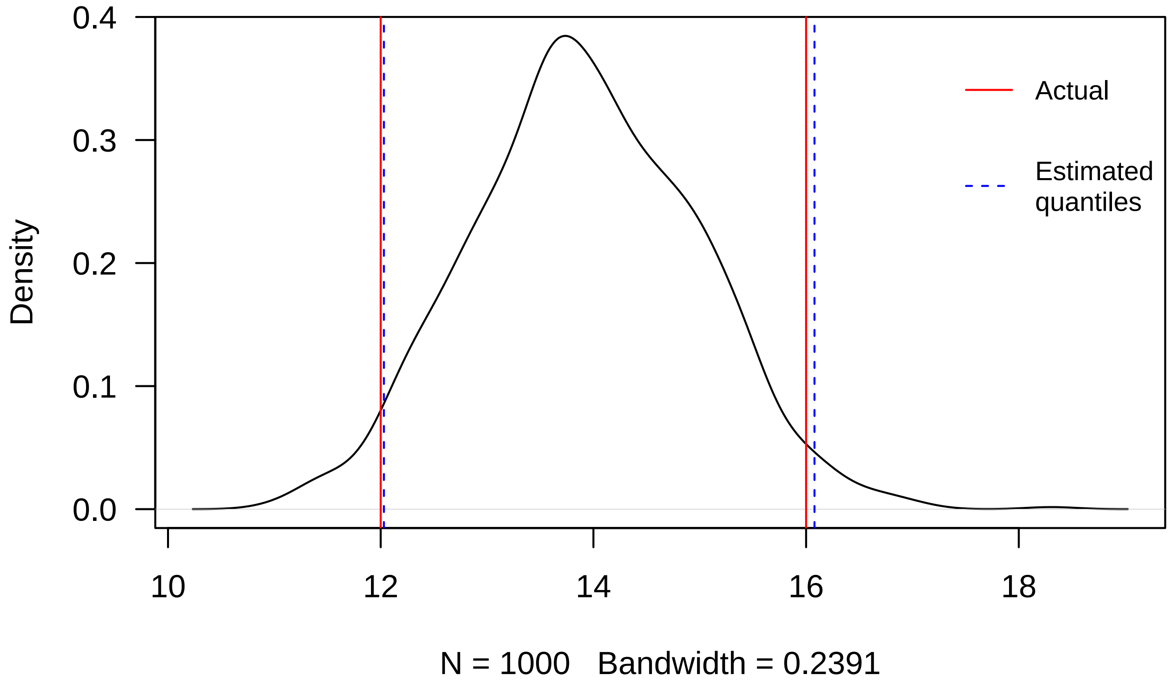
0.4
0.3
0.2
0.1
0.0
Density
Actual
Estimated
quantiles
10
12
14
16
18
N = 1000 Bandwidth = 0.2391

Interaction models

Context

This is a set of complementary analyses designed to test for sex-specific differences in the slope parameters of linear measurements used to predict total length and carapace length in buthid scorpions. To assess these differences, an interaction term between sex and each predictor was added to the models. The regression models were built using a Bayesian framework with the R package `MCMCglmm` (Hadfield 2010) accounting for the phylogenetic relationships among species. In essence, these are phylogenetic mixed linear models fitted for trait data with repeated measures (i.e., the species), which were used as the random factor in the analyses. The dataset included natural logarithm values of linear measurements from 195 species. Abbreviations for the traits are provided in the table below.

Trait abbreviation:

Trait	Abbreviation
Total length	ToL
Carapace length	CarL
Chela length	CheL
Chela width	CheW
Length of metasomal segment V	Met5L
Width of metasomal segment V	Met5W
Telson length	TeL
Telson width	TeW

Interactions with sex were not statistically significant when predicting total length

The table below presents the interaction terms between sex and each predictor, using total length as the response variable. The models were specified with species included as a random factor to account for repeated measures (i.e., one measurement per sex). All analyses considered the phylogenetic dependence of trait values (see the main text for further details). ESS = effective sample size.

Model	Parameter	Posterior Mean	Lower 95% CI	Upper 95% CI	ESS
M1	Intercept	2.1067	1.9907	2.2011	1000
	carL	1.0333	0.9881	1.0772	900
	sexm	0.0367	-0.0295	0.1062	816
	carL:sexm	0.0149	-0.0247	0.0520	863
M2	Intercept	2.3406	2.1305	2.5767	900
	cheL	0.6968	0.6367	0.7581	999
	sexm	-0.0847	-0.1700	0.0055	900
	cheL:sexm	0.0326	-0.0060	0.0728	900
M3	Intercept	3.4082	3.2306	3.5734	836
	cheW	0.6405	0.5808	0.7105	900
	sexm	-0.0148	-0.0531	0.0245	900
	cheW:sexm	-0.0204	-0.0626	0.0254	900
M4	Intercept	2.1614	2.0517	2.2747	900
	met5L	0.9480	0.9016	0.9922	900
	sexm	-0.0424	-0.1040	0.0241	900
	met5L:sexm	-0.0158	-0.0481	0.0214	900
M5	Intercept	3.1300	2.9011	3.3314	900
	met5W	0.7158	0.6437	0.7938	1002
	sexm	0.0097	-0.0448	0.0677	900
	met5W:sexm	-0.0206	-0.0746	0.0258	900
M6	Intercept	2.3378	2.2198	2.4567	940
	teL	0.8932	0.8516	0.9409	900
	sexm	-0.0261	-0.0847	0.0336	900
	teL:sexm	0.0166	-0.0192	0.0507	900
M7	Intercept	3.2794	3.1353	3.4318	992
	teW	0.8140	0.7608	0.8611	900
	sexm	0.0005	-0.0300	0.0275	900
	teW:sexm	0.0279	-0.0041	0.0646	900

Interactions with sex were mostly non significant when predicting carapace length

The table below presents the interaction terms between sex and each predictor, using carapace length as the response variable. The models were specified with species included as a random factor to account for repeated measures (i.e., one measurement per sex). All analyses considered the phylogenetic dependence of trait values (see the main text for further details). Only the interaction with metasomal segment V (`ln_met5L:sexm`) was statistically significant. ESS = effective sample size.

Model	Parameter	Posterior Mean	Lower 95% CI	Upper 95% CI	ESS
M1	Intercept	0.4280	0.1693	0.6841	996
	cheL	0.5755	0.5257	0.6271	950
	sexm	-0.1089	-0.1644	-0.0581	900
	cheL:sexm	0.0136	-0.0087	0.0404	900
M2	Intercept	1.2761	1.1072	1.4480	900
	cheW	0.5668	0.5081	0.6272	900
	sexm	-0.0656	-0.1014	-0.0363	805
	cheW:sexm	-0.0337	-0.0696	0.0005	900
M3	Intercept	0.2023	0.0624	0.3322	900
	met5L	0.8267	0.7681	0.8760	900
	sexm	-0.0493	-0.1160	0.0234	900
	met5L:sexm	-0.0420	-0.0755	-0.0001	900
M4	Intercept	1.0414	0.8626	1.2410	900
	met5W	0.6400	0.5778	0.6975	900
	sexm	-0.0725	-0.1088	-0.0367	900
	met5W:sexm	0.0006	-0.0321	0.0315	900
M5	Intercept	0.3500	0.2297	0.4848	1083
	teL	0.7830	0.7414	0.8263	1212
	sexm	-0.0804	-0.1240	-0.0290	862
	teL:sexm	0.0107	-0.0160	0.0378	874
M6	Intercept	1.1788	1.0365	1.3087	900
	teW	0.7154	0.6698	0.7572	793
	sexm	-0.0591	-0.0795	-0.0379	818
	teW:sexm	0.0205	-0.0036	0.0467	900

Phylogenetic multiple regressions

Context

The results of several multiple regression models analyzed using a Bayesian phylogenetic approach are presented below. These models were designed to evaluate whether including a secondary predictor improves the accuracy of predictions for total length and carapace length in buthid scorpions - remember that in the previous analyses (main text), `Met5L` and `TeL` appeared as the best single predictor (lowest RMSE values) of total length and carapace length, respectively. The multiple regression models were built using a Bayesian framework with the R package `MCMCglmm` (Hadfield 2010) accounting for the phylogenetic relationships among species. In essence, these are phylogenetic mixed linear models fitted for trait data with repeated measures (i.e., the species), which were used as the random factor in the analyses. The dataset included natural logarithm values of linear measurements from 195 species.

Multiple regression for predicting total length

The multiple regression analyses revealed that while adding a second predictor improves the accuracy of total length predictions, the improvement is modest. The RMSE for the model using `Met5L` as the sole predictor was 6.42 mm, corresponding to an average relative error of 13% (with the mean total length in the dataset being 51.28 mm). In comparison, the RMSE for the best multiple regression model, which combines `Met5L` with `CheW`, is 5.13 mm, leading to an average relative error of 10%. Therefore, the addition of a second predictor enhances the accuracy of total length predictions by only 3% at most. ESS = effective sample size.

Model	RMSE (mm)	Parameter	Posterior Mean	Lower 95% CI	Upper 95% CI	ESS
M4	5.129	Intercept	2.331	2.245	2.413	900
		met5L	0.767	0.722	0.820	900
		cheW	0.196	0.157	0.233	900
M3	5.147	Intercept	2.497	2.393	2.614	1066
		met5L	0.619	0.558	0.681	1045
		teW	0.344	0.279	0.395	992
M5	5.220	Intercept	2.235	2.137	2.330	900
		met5L	0.783	0.729	0.833	900
		met5W	0.204	0.157	0.251	900
M1	5.451	Intercept	2.165	2.063	2.247	900
		met5L	0.472	0.400	0.552	900
		teL	0.491	0.415	0.561	900
M2	5.637	Intercept	2.041	1.936	2.130	900
		met5L	0.739	0.683	0.803	900
		cheL	0.218	0.170	0.269	900

Multiple regression for predicting carapace length

Similarly, for the multiple regression models predicting carapace length, adding a secondary predictor improved accuracy by only up to 2% at most. The mean carapace length in the dataset was 5.6 mm. ESS = effective sample size.

Model	RMSE (mm)	Parameter	Posterior Mean	Lower 95% CI	Upper 95% CI	ESS
M5	0.559	Intercept	0.337	0.249	0.432	665
		teL	0.653	0.602	0.703	900
		met5W	0.224	0.173	0.273	900
M3	0.571	Intercept	0.576	0.465	0.680	900
		teL	0.493	0.425	0.561	900
		teW	0.362	0.296	0.428	900
M4	0.577	Intercept	0.390	0.306	0.484	817
		teL	0.681	0.633	0.733	900
		cheW	0.172	0.134	0.220	900
M2	0.622	Intercept	0.147	0.046	0.252	900
		teL	0.702	0.640	0.760	900
		cheL	0.150	0.102	0.210	900
M1	0.656	Intercept	0.188	0.071	0.282	900
		teL	0.727	0.647	0.815	900
		met5L	0.131	0.035	0.219	900

References

Esposito, L. A., & Prendini, L. (2019). Island ancestors and new world biogeography: A case study from the scorpions (Buthidae: Centruroidinae). Scientific Reports, 9(1), 3500.

Hadfield J. D. (2010). MCMC Methods for Multi-Response Generalized Linear Mixed Models: The MCMCglmm R Package. Journal of Statistical Software, 33(2), 1-22. URL https://www.jstatsoft.org/v33/i02/.

Ojanguren-Affilastro, A. A., Adilardi, R. S., Mattoni, C. I., Ramírez, M. J., & Ceccarelli, F. S. (2017). Dated phylogenetic studies of the southernmost American buthids (Scorpiones; Buthidae). Molecular Phylogenetics and Evolution, 110, 39–49. https://doi.org/10.1016/j.ympev.2017.02.018
